# Citric acid is more effective than sodium thiosulfate in chelating calcium in a dissolution model of calcinosis

**DOI:** 10.1038/s41598-024-65761-3

**Published:** 2024-12-28

**Authors:** Kyle A. Burgess, Richard E. P. Winpenny, Alberto Saiani, Aline F. Miller, Ariane L. Herrick, Rachel E. B. Watson

**Affiliations:** 1 Division of Musculoskeletal and Dermatological Sciences, School of Biological Sciences, Faculty of Biology, Medicine and Health, The University of Manchester and Northern Care Alliance NHS Foundation Trust, Manchester Academic Health Science Centre, Manchester, M13 9PT UK; 2 Department of Chemistry, The University of Manchester, Manchester, UK; 3 Department of Materials, The University of Manchester, Manchester, UK; 4 Manchester Institute of Biotechnology, Department of Chemical Engineering, School of Engineering, The University of Manchester, Manchester, UK; 5 NIHR Manchester Biomedical Research Centre, Manchester University NHS Foundation Trust, Manchester Academic Health Science Centre, Manchester, UK; 6 A*STAR Skin Research Labs (A*SRL), Agency for Science, Technology and Research (A*STAR), Singapore, Republic of Singapore

**Keywords:** Rheumatology, Chemistry

## Abstract

Calcinosis cutis affects 20–40% of patients with systemic sclerosis. This study tests the hypothesis that calcium-chelating polycarboxylic acids can induce calcium dissolution without skin toxicity or irritancy. We compared citric acid (CA) and ethylenediaminetetraacetic acid (EDTA) to sodium thiosulfate (STS) for their ability to chelate calcium in vitro using a pharmaceutical dissolution model of calcinosis (hydroxyapatite (HAp) tablet), prior to evaluation of toxicity and irritancy in 2D in vitro skin models. Resultant data was used to predict therapeutic concentrations for application in a validated 3D skin irritation model (SkinEthic™; EpiSkin SA) and to assay maximal percutaneous absorption. Dissolution performance was further assessed via ability to dissolve a calcified matrix laid down in vitro. Pharmacological dissolution studies identified that polycarboxylic acids were superior to STS in dissolving HAp tablets. In vitro, compounds had little effect on cell numbers at concentrations of < 10 mM. When applied topically to 3D models as near-saturated solutions, chelators were not irritant nor did they impact model structure histologically. CA was the most efficient chelator of calcium salts. This study highlights polycarboxylic acids, particularly CA, as potential therapies to target calcinosis cutis: these should now be investigated in human studies.

## Introduction

**Figure 1:**
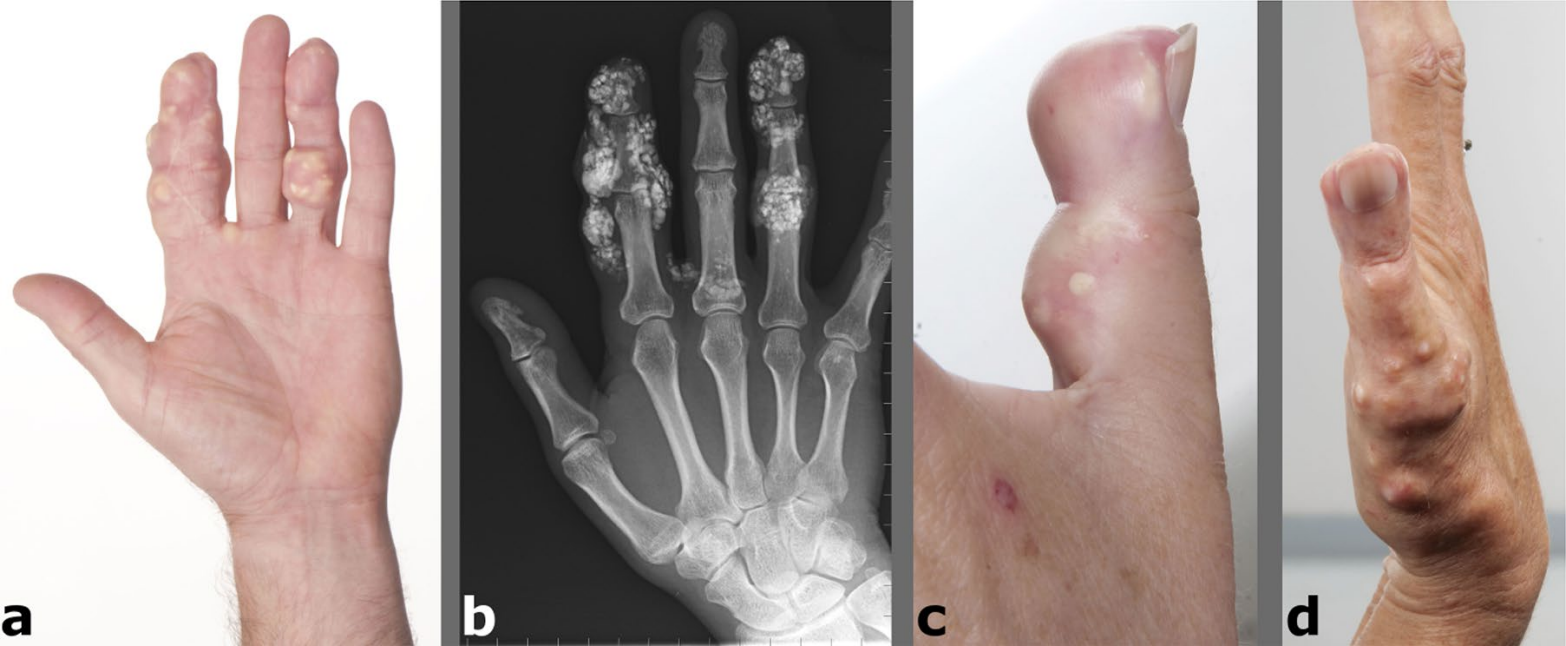
Images depicting examples of SSc-related calcinosis. *Calcinosis cutis* in the first and third digits of the hand (**a**,**b**), the calcinosis is well demonstrated on plain radiography (**b**), and the thumb (**c**,**d**). Images copyright of Northern Care Alliance NHS Foundation Trust.

Calcinosis (sub-epidermal deposition of calcium salts; mainly hydroxyapatite, HAp)^1^ is a major clinical problem in patients with connective tissue disorders, in particular systemic sclerosis (SSc) where it affects ~ 20–40% of patients. For these individuals, calcinosis represents an area of significant unmet clinical need^2,3^. Calcinosis typically affects the hands (~ 70% of cases), especially the fingers and thumbs (Fig. 1), but can develop at other upper and lower limb sites (especially over pressure points), and (less commonly) on the face, neck and trunk^4^. Calcinosis is often painful, disabling, disfiguring, and at worse, life-threatening if it leads to systemic infection^5^. The pathogenesis of calcinosis, including SSc-related calcinosis, remains unknown^3,4,6^, thereby hindering any development of disease-modifying pharmacotherapy. As such, there is currently no effective treatment. Instead, disease management strategies comprise: (1) medical care, mainly prompt treatment of superadded infections and analgesia and; (2) surgical care (debridement or ‘debulking’)^7^.

To better manage calcinosis, a promising strategy is topical application of calcium-chelating compounds which penetrate the epidermal barrier and dissolve subcutaneous calcium deposits^8^. In general, localised application of drug(s) to affected skin areas is less likely to cause systemic side effects than oral or intravenous delivery. In the case of SSc-related calcinosis, lesions are often ischaemic^9^ which may further reduce the efficacy of systemically delivered treatments. Hence, topical application may support achieving higher concentration of the compound(s) at the site(s) of interest, so providing effective dissolution of calcinotic deposits.

A small number of reports have claimed some clinical improvement following topical application of sodium thiosulfate (STS) when treating cases of refractory, non-healing calcinosis^10–13^; STS was tried topically based on its previous licenced use for calciphylaxis^14^. Calcium displaces sodium in STS to form calcium thiosulfate, a much more soluble calcium salt. However, others argue STS inhibits vascular calcification independent of interactions with calcium or hydroxyapatite^15^. As of 2015, the potential use of STS as a topical therapy in ectopic calcification has led to filing of active patents in Europe, the USA and Japan (WO2013167741A1) and an observational study involving 120 patients (ClinicalTrials Identifier: NCT03979378). However, the efficacy of topical STS has been challenged in a recent retrospective study (2012–2017) where only partial dissolution of ectopic calcification resulted^16^. A recent case study suggests the success of topical STS was found to be largely dependent on lesion size: complete resolution was achieved for all calcinotic lesions < 0.2 cm in size, whereas the success rate dropped to 78% and 20% for larger lesions initially categorised as between 0.2 to 0.3 and 0.3 to 0.5 cm respectively^17^.

In a bid to develop more effective topical therapies for calcinosis one strategy is to explore other calcium-chelating compounds for their relative effectiveness at dissolving subcutaneous calcification. Of particular interest are the polycarboxylic acids citric acid (CA) and ethylenediaminetetraacetic acid (EDTA). Both compounds are used at low concentrations in dermal preparations as pH adjusters and/or chelating agents^18,19^. At much higher concentrations, these compounds are used routinely in dentistry to remove the smear layer during root canal surgery^20^. Due to their calcium-chelating properties, both compounds have been recently implicated as potential therapeutic agents for calcinosis dissolution^21^.

This study provides the first in vitro comparison of different calcium chelators (STS, CA and EDTA) as topical agents aimed at managing calcinosis. Efficacy of calcium salt dissolution was quantified, as was their potential to induce skin irritation in both 2D (keratinocyte and fibroblast) and 3D skin-equivalent (SkinEthic™) models, the latter providing a reliable measure of maximum therapeutic concentration and relative skin absorptivity. At predicted ‘dermal’ concentrations, the relative efficacy of each compound was confirmed using dissolution of both HAp and a calcified in vitro model of calcinosis.

## Materials and methods

### Materials

Compounds (STS, CA and EDTA disodium salt; ≥ 99% purity) were purchased from Sigma Aldrich (Dorset, UK). For cell culture experiments, these were prepared in 100 mM HEPES buffer (pH 7.4) at 10× stock concentrations which were then filter-sterilised and dissolved in cell culture media (10% v/v) before use. For skin-equivalent models, compounds were prepared in 10 mM HEPES buffer (pH 5.5), filter-sterilised and used neat. SkinEthic™ models (RHE/S/17: reconstructed human epidermis, small, age day 17–0.5 cm^2^) and nylon meshes (0.5 cm^2^) were purchased from EPISKIN (Lyon, France). All other materials and reagents were purchased from Thermo Fisher Scientific (Loughborough, UK), unless stated otherwise.

### Pharmaceutical dissolution of hydroxyapatite tablet

Pharmaceutical dissolution of HAp was modelled as previously described^21^ (supplemental information). All dissolution studies were performed in triplicate. A vehicle control was used to account for any non-specific dissolution. Calcium concentrations were expressed as HAp based on the relationship that 1 mol calcium = 100.5 g HAp. Dissolution was assessed after 1-h and 5-h, dependent on the experiment.

### Cell culture

A spontaneously immortalized human keratinocyte line (HaCaT) was maintained under standard cell culture conditions (37 °C, 5% CO_2_) using Dulbecco's modified Eagle's medium (DMEM; Invitrogen, Paisley, UK) supplemented with 10% foetal bovine serum (FBS; Invitrogen), 1% GlutaMAX™ (100×; Invitrogen) and 1% penicillin/streptomycin solution (100×; Invitrogen). HaCaT cells were purchased from CLS Cell Lines Service GmbH (Eppelheim, Germany). At 70–80% confluence, cells were sub-cultured using TrypLE cell dissociation reagent and used in downstream experiments, as specified. Similarly, a human primary osteogenic sarcoma cell line (SaOS-2; a kind gift from Dr. Steven Richardson, University of Manchester) was maintained as above using DMEM supplemented with 10% FBS, 1% penicillin/streptomycin solution (100×) and fresh 50nM ascorbic acid. SaOS-2 cells were purchased from Sigma (Sigma-Aldrich, UK).

### Cell viability

Relative cell viability was determined using the alamarBlue™ assay, a metabolic assay which indicates the number of viable cells based on their ability to reduce a cell-permeable reagent [resazurin] into a fluorescent product [resorufin]. Cells were seeded at a density of 15,625 cells/cm^2^. After 4-h, cells were treated with increasing concentrations (0.0001–10 mM) of test compounds (STS, CA and EDTA) for either 2- or 24-h. Control samples were incubated with 10 mM HEPES, pH 7.4 (vehicle). Media was removed and plates were washed with phosphate buffered saline (PBS; 137 mM NaCl, 2.7 mM KCl, 8 mM Na_2_HPO_4_, 2 mM KH_2_PO_4_) to avoid any non-specific reduction by the compounds^22^. Cells were then incubated with fresh media containing alamarBlue™ reagent (10% v/v) for 1-h. Relative fluorescence (540 nm/590 nm) was measured (FLx800 fluorescent plate reader; Agilent Technologies; Stockport, UK). A sample without cells was used to subtract any background signal. For viability recovery experiments, calcium chloride was dissolved in cell culture media and filter-sterilised before the addition of test compounds.

The percentage of necrotic cells was determined using the lactate dehydrogenase (LDH) assay. Cells were seeded as above and treated with EDTA (10 mM) for 2-h. Positive and negative controls were prepared by treating cells with lysis buffer or vehicle, respectively. After 2-h, cell culture plates were centrifuged, media sampled and then mixed with LDH reagent for 25 min under constant agitation (photoprotected, at room temperature). Relative absorbance was measured at 490 nm using a plate reader (CLARIOstar, BMG LABTECH; Aylesbury, UK) and adjusted by subtracting the background at 680 nm.

### Gene expression analysis

Relative gene expression was determined using reverse transcription quantitative polymerase chain reaction (RT-qPCR). In brief, cells were cultured to a confluence of 70–80% before treating with test compounds (10% v/v; diluted in media). After 3-h, total RNA was extracted using the RNeasy Mini Kit (Qiagen; Manchester, UK) and purified with amplification grade DNase I (Sigma Aldrich). DNase-treated RNA was transcribed into cDNA using a high-capacity RNA-to-cDNA kit supplemented with RNase inhibitor, as per manufacturer's instructions. For RT-qPCR, cDNA (10 ng per reaction) was amplified using the Brilliant III Ultra-Fast SYBR1 QPCR kit (Agilent Technologies) following a two-step RT-qPCR protocol (enzyme activation for 3 min at 95 °C, followed by 40 cycles of denaturation (95 °C) and annealing/elongation (60 °C) for 5 and 25 s, respectively). All reactions were performed in triplicate using pre-designed primers: glyceraldehyde 3-phosphate dehydrogenase (GAPDH); interleukin (IL)-1α; and IL-8 (QuantiTect; Qiagen). RT-qPCR was performed on a StepOnePlus™ Real-Time PCR System (Applied Biosystems). The expected product sizes were confirmed by melting temperature (Tm) analysis in the range 60–95 °C; all samples produced identical, overlapping melting curves with a single peak at the expected Tm value.

### Quantification of protein secretion by ELISA

Secreted protein was determined using enzyme-linked immunosorbent assay (ELISA) for IL-1α and IL-8. Briefly, cells were treated as described previously for gene expression analysis. After 24-h, the media was collected, centrifuged and supernatant stored at − 80 °C. Secreted protein was quantified using DuoSet^®^ ELISA Development Kits (R&D Systems; Oxford, UK), as per manufacturer’s instructions.

### SkinEthic™ skin irritation testing (SIT)

Skin irritation was assessed according to the SkinEthic™ Irritation Test (SIT: DB-ALM Protocol #135, EpiSkin)^23^. Briefly, solutions of each compound (32 μL/cm^2^) were applied topically to SkinEthic™ models (Day 17) for 42 ± 1 min at room temperature, followed by an incubation period of 42 ± 1 h (37 °C, 5% CO_2_). Each compound was tested at a range of concentrations approaching maximum solubility, as follows: STS, 200–1200 mM; CA, 200–1600 mM; and EDTA, 50–200 mM. In each case, the dilution series was prepared according to their relative maximum solubility in water. After compound application, a nylon mesh (0.5 cm^2^) was placed on top of the model to ensure even coverage of test solution. Models were also treated with the following to provide appropriate controls: vehicle—HEPES (10 mM, pH 5.5); negative—PBS (Ca^−^/Mg^−^); positive—5% sodium dodecyl sulfate (SDS; prepared using de-ionised water). Tissue viability was assessed using the MTT assay. If viability < 50%, compounds are considered Category 2 skin irritants. For STS-treated tissues, the irritation test was repeated using water-killed SkinEthic™ models to check for any background from non-specific reduction of MTT. No measurable amount of background signal was detected.

### SkinEthic™ compound absorption and histological analysis

Relative skin absorptivity was measured using SkinEthic™ models. Briefly, a solution of each compound was applied topically (32 μL/cm^2^) to the model, as described above. Each compound was prepared fresh at the maximum concentration shown not to induce an irritation response. The SkinEthic models were incubated at room temperature for 1 h prior to applying each compound, and then absorption studies were conducted at room temperature for up to 4 h. At each time point (30 min, 1-, 2- or 4-h), the receptor media was collected and the amount of compound was determined using ion chromatography (supplemental information). The rate of compound absorption (0.5–4-h) was calculated using a linear regression model.

At each time-point (30 min, 1-, 2- or 4-h), the model was fixed in neutral-buffered formalin (10%) for 24-h at 4 °C. Models were dehydrated, clearing in xylene and wax impregnation (Miles Sakura Tissue Tek VIP 2000; Newbury, UK). Tissue sections were cut (5 μm), re-hydrated and stained with haematoxylin and eosin (H&E); images were captured using a BX53 microscope (Olympus Industrial; Southend-on-Sea, UK).

### Calcified extracellular matrix model

SaOS-2 cells were seeded in 6-well plates (10,000 cells/cm^2^), cultured for 5 days until 100% confluent and maintained in osteogenic media for a further 7–9 days (DMEM: 0.5% FBS, penicillin/streptomycin solution (100×), 10 mM glycerophosphate, 10 nm dexamethasone, 50 μg/mL fresh ascorbic acid). Calcified matrices were fixed in situ with 4% paraformaldehyde and washed twice with HEPES buffer prior to addition of chelator solutions (1 mL). Plates gently agitated at room temperature for between 5 min to 6-h. At specified time points, chelator solutions were removed and analysed using the phosphate quantification assay (AbCam; Cambridge, UK). The calcified matrices were washed twice with ddH_2_O and the extent of calcification remaining was analysed using the alizarin red S (ARS) quantification assay (ScienCell/Caltag MedSystems; Buckingham, UK).

### Statistical analysis

All data are presented as mean ± standard deviation, unless otherwise stated. Comparisons between two groups were performed using a Student’s *t*-test. For multiple comparisons, statistical analysis was performed using a one-way ANOVA and Dunnett’s post-hoc test comparing each condition to the relative control sample (e.g., untreated or vehicle). Statistical significance was determined when P < 0.05. Statistical analysis was performed using GraphPad Prism software (California, USA).

## Results

### Polycarboxylic acids achieve superior calcium chelation in vitro than current topical treatments

Dissolution of a HAp tablet—a reproducible, synthetic mimic of calcinosis^21^—was used to test the relative dissolution performance of compounds after 1-h of exposure (N = 3). Compounds could be ranked in terms of their relative ability to dissolve HAp at physiological pH; EDTA (37.4 ± 0.9 mg h^−1^) > CA (9.0 ± 0.9 mg h^−1^) > STS (0.3 ± 0.2 mg h^−1^). Both polycarboxylic acids performed better than STS, with EDTA and CA dissolving > 100× and 30× the amount of HAp compared to STS, respectively. Interestingly, STS did not perform better than the vehicle control (1.8 ± 0.3 mg h^−1^).

### Polycarboxylic acid treatment does not result in cell death in vitro but disrupts calcium-dependent cell–cell connectivity

**Figure 2:**
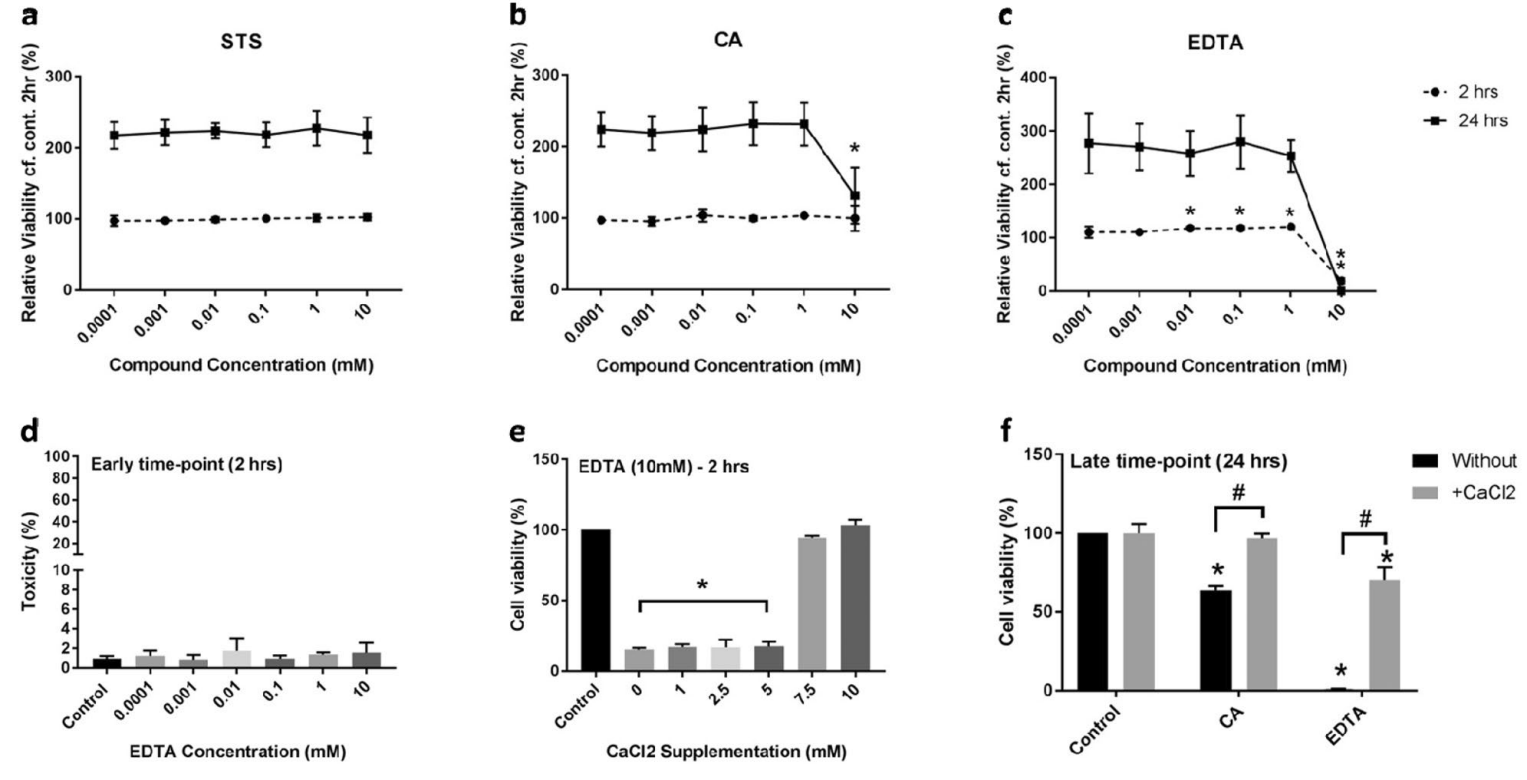
Impact of calcium chelation on cell viability. HaCaT cells were seeded into 96-well plates. After 4-h, cells were treated with increasing concentrations (0.0001–10 mM) of calcium-chelating compounds: (**a**) STS, (**b**) CA and (**c**) EDTA, for 2-h (dotted line) and 24-h (solid line). All viability measurements are relative to the control sample (vehicle) at 2-h. (**d**) Percentage of necrotic cells following incubation with increasing concentrations of EDTA (0.0001–10 mM) for 2-h. The relative amount of LDH in the supernatant was determined for each sample and compared to cells treated with lysis buffer (positive control). (**e**) Percentage of viable HaCaT cells following treatment with EDTA (10 mM; 2-h) in the presence of increasing concentrations of CaCl_2_ (0–10 mM). (**f**) Percentage of viable HaCaT cells following treatment with CA (10 mM) or EDTA (10 mM) for 24-h, with/without additional CaCl_2_ supplementation (10 mM). Cell viability was determined using the alamarBlue assay. In all cases, data is presented as mean ± stdev (N = 3). Significance was determined either relative to the vehicle control (*), or between conditions as highlighted (^#^); P < 0.05. *STS* sodium thiosulfate, *CA* citric acid, *EDTA* ethylenediaminetetraacetic acid.

Cell numbers were unaffected by treatment with STS (≤ 10 mM) (Fig. 2a). When cells were treated with CA or EDTA, a reduction in cell number (cytotoxicity) was only observed when testing compounds at their maximal concentration (10 mM; Fig. 2b,c), with EDTA having a more immediate effect than CA (2-h vs. 24-h). As calcium is essential for keratinocyte adhesion^24^, changes to cell numbers may be due to calcium-chelation alone. Hence, cell detachment *vs.* toxicity (cell lysis) was assessed via relative levels of LDH in the supernatant. Following EDTA treatment (0.0001–10 mM), we observed no significant increase in the percentage of lysed cells *cf.* control (vehicle) (Fig. 2d). Therefore, the decrease in cell numbers appeared to be due to cell detachment as opposed to cell death. To confirm this, cell viability measurements were repeated in the presence of additional calcium chloride (CaCl_2_). Cell viability was measured after 2-h, relative to control (vehicle); when concentrations ≥ 7.5 mM CaCl_2_ were added back into the media, cell detachment was abolished (Fig. 2e). Cell viability measurements were also determined at 24-h post-treatment with both 10 mM CA and EDTA in the presence of additional CaCl_2_ (10 mM). When the media was supplemented with CaCl_2_, cell loss was mitigated (Fig. 2f).

### Calcium chelation does not induce expression of inflammatory mediators or induce irritation in skin model systems

To ensure that chelators did not elicit a pro-inflammatory response when applied to skin, we assessed cytokine expression in HaCaT cells and in the RHE (SkinEthic™) model. HaCaT cells were treated with increasing concentrations of each compound and assessed for downstream gene (3-h) and protein (24-h) expression of the cytokines IL-1α and IL-8, accepted biomarkers of an acute skin irritation response^25^. Lipopolysaccharide (LPS) was added to cultures as a positive control^26^. As expected, LPS (10 μg/mL) significantly upregulated both IL-1α and IL-8 gene expression and subsequent IL-8 protein secretion *cf.* control (Supplementary Fig. S1a, S1b). However, IL-1α was not detected in culture supernatant regardless of treatment [data not shown]. Importantly, treatment with LPS (≤ 10 μg/mL) did not induce cytotoxicity. Neither IL-8 gene expression (Supplementary Fig. S1a) nor protein secretion (Supplementary Fig. S1b) *cf.* control was observed following chelator treatment at the concentration range tested (0.1–10 mM). Interestingly, cells treated with 10 mM CA or EDTA significantly reduced their relative secretion of IL-8 *cf.* untreated control (Supplementary Fig. S1b). Specifically, IL-8 was reduced to 53.9 ± 3.2% and 40.9 ± 10.6% in the presence of CA and EDTA, respectively. To better gauge relative therapeutic concentrations, compounds were screened using the SkinEthic™ Skin Irritation Test (SIT). At solution concentrations approaching maximum solubility (STS—1200 mM [19% w/v]; CA—1600 mM [31% w/v]; EDTA—200 mM [7.5% w/v]), none of the chelators were classified as skin irritants (Supplementary Fig. S2a–c). For all concentrations tested, all compounds resulted in > 50% viability (red dotted line). Hence, saturated solutions of any of the compounds should not induce skin irritation in vivo.

### Citric acid (CA) is better absorbed through RHE models than either EDTA or STS, resulting in improved cumulative exposure

**Figure 3:**
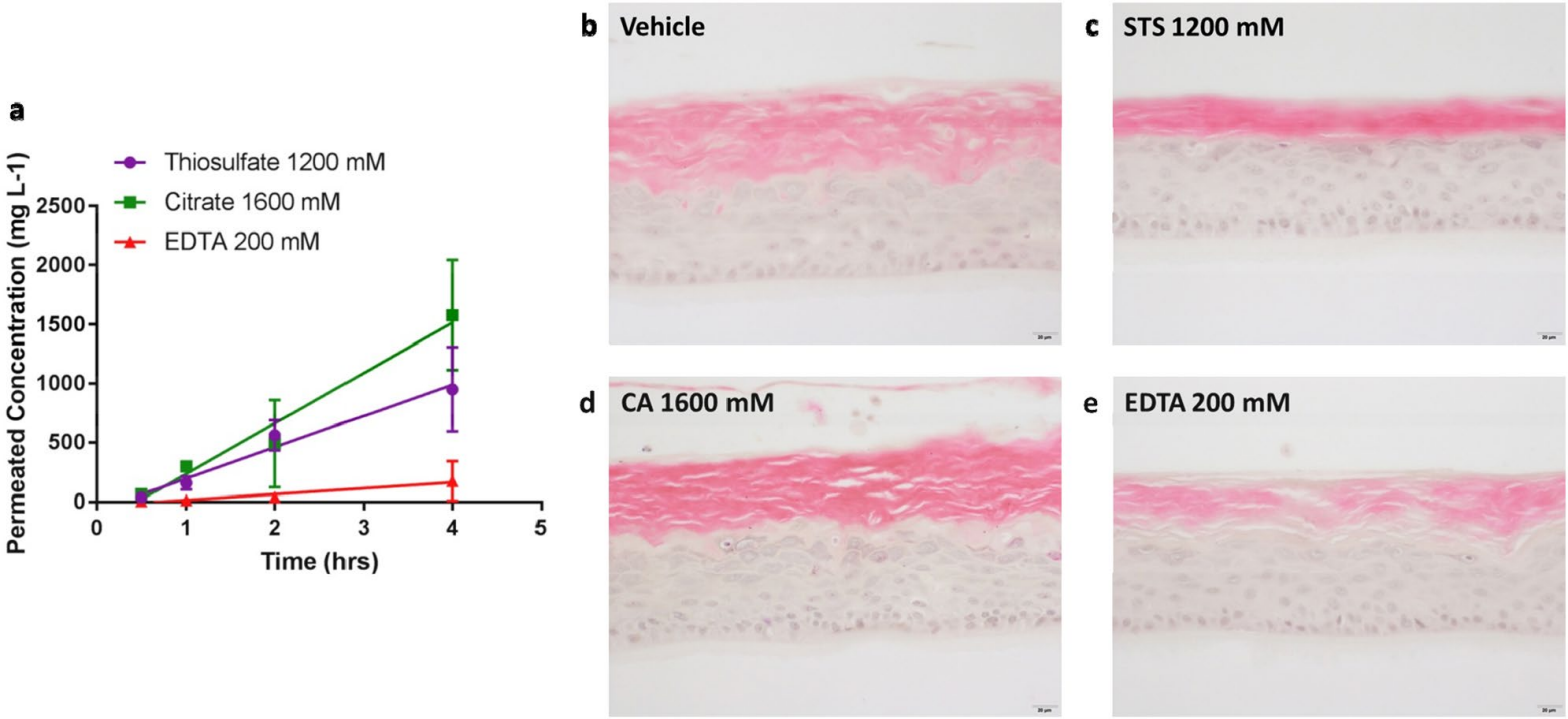
Percutaneous absorption of calcium chelators using recombinant human epidermal models (SkinEthic™). SkinEthic™ models (day 17) were treated with STS (1200 mM), CA (1600 mM) or EDTA (200 mM), at pH 5.5 (10 mM HEPES buffer), for up to 4-h at room temperature. (**a**) At different time points, the receptor media was collected and the concentration of compound was quantified using ion chromatography. Data is presented as mean ± stdev (N = 4) of the absolute value. (**b**–**e**) Representative images of models following treatment with each of the calcium chelators for 4-h. Images are taken at ×20 magnification (scale bar 20 μm).

To assess maximum penetration concentration, solutions of each compound were applied to SkinEthic™ models using the same experimental procedure as the SIT (finite-dose). Compound concentrations in the receptor media were then quantified and plotted as a factor of time. Following topical application, all three compounds were detected in the receptor media after 30 min (Fig. 3a). Over the course of 4-h, the rate of absorption was proportional to the time of incubation. Using a linear regression model, the rate of compound absorption was calculated as: CA (1600 mM), 0.43 ± 0.05 g/L/h; STS (1200 mM), 0.26 ± 0.03 g/L/h and EDTA (200 mM), 0.05 ± 0.01 g/L/h. At each time-point, the cumulative concentration of compound in the receptor media was CA > STS > EDTA. These rankings correlate with the initial concentration of compound applied.

At each time-point, the RHE model was fixed and stained with haematoxylin and eosin to assess epidermal morphology. At 4-h post-application, the structure of the epidermis remained intact and comparable to the vehicle control following application of all chelators (Fig. 3b–e). Topical application to the RHE model with maximal concentrations of CA or EDTA did not alter cell-to-cell interactions within any of the viable cell layers.

### Pharmacological dissolution studies identify the superiority of polycarboxylic acids over STS in dissolving of hydroxyapatite

**Figure 4:**
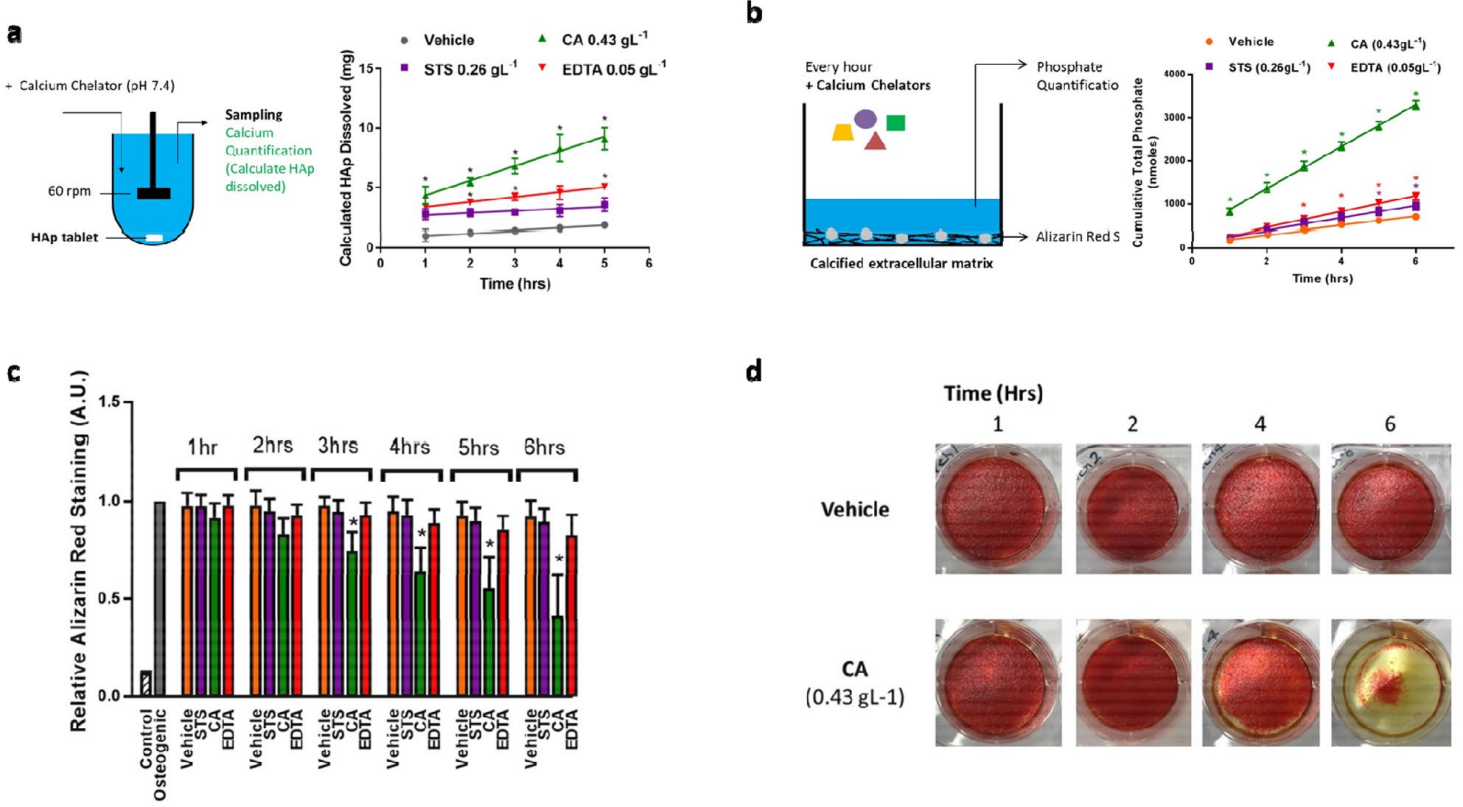
Pharmacological dissolution of hydroxyapatite in vitro. (**a**) Schematic representation of a pharmaceutical dissolution approach using a tablet of hydroxyapatite. Every hour, a sample was collected and the amount of calcium ions was quantified as a measure of hydroxyapatite dissolution. The amount of hydroxyapatite dissolved per hour is shown for each calcium chelator (STS, CA and EDTA) over a 5-h time course. The concentration of chelator tested was calculated from the rate of absorption through SkinEthic™ models, which was 0.26, 0.43 and 0.05 g/L for STS, CA and EDTA, respectively. Data is presented as mean ± stdev (N = 3); *P < 0.05 cf. STS. (**b**) Schematic representation of a calcified matrix model. Every hour, supernatants were collected and fresh chelator solution added. Calcification dissolution was determined by measuring: (1) the total amount of phosphate in solution, and (2) staining the matrix for remaining calcification using Alizarin Red S. The cumulative phosphate concentration is plotted as a factor of time. (**c**) For each time point, the amount of Alizarin Red S is quantified relative to untreated calcified matrix (osteogenic). As a negative control, cells were also cultured in normal media without osteogenic stimulation (control). Data is presented as mean ± stdev (n = 3); *P < 0.05 compared to vehicle. (**d**) Representative images of Alizarin Red S staining for the vehicle and CA at multiple time points (1–6 h). The concentration of chelator tested was calculated from the rate of absorption through SkinEthic™ models, which is: 0.26, 0.43 and 0.05 g/L for STS, CA and EDTA, respectively. Data is presented as mean ± stdev (N = 3).

Having identified that maximal chelator concentrations did not result in deleterious effects on the RHE model, pharmaceutical dissolution was repeated. Samples were collected at multiple time points (1- and 5-h) and dissolution measured by ion chromatography (Fig. 4a). At all time-points, chelator treatment resulted in significantly greater HAp dissolution *cf.* vehicle control. The cumulative amount of HAp dissolved into solution remained proportional to treatment time for all chelators tested, in the order CA > EDTA > STS. After 5-h, the total amount of HAp dissolved into solution was: CA, 9.1 ± 0.1 mg, EDTA, 5.1 ± 0.3 mg, STS, 3.6 ± 0.6 mg with CA solution reproducibly resulted in significantly more HAp dissolution *cf.* STS solution. After 1-h, no significant difference in HAp dissolution was observed between EDTA and STS treatment. However, significantly more HAp was dissolved after 2-h of treatment with EDTA *cf.* STS solution. Whilst EDTA proved most effective at dissolving HAp when tested at 10 mM, CA solution proved more effective *cf.* EDTA as higher concentration could be achieved in solution (0.43 vs. 0.05 g/L). Interestingly, EDTA still proved more effective at dissolving HAp *cf.* STS, despite being used at much lower concentrations (0.26 vs. 0.05 g/L).

### Deliverable concentrations of CA show superior dissolution of an in vitro calcified matrix than EDTA or STS

To further assess relative therapeutic efficacy in vitro, compounds were tested on monolayers of calcified matrix. After 1-h, significantly more phosphate was detected in the supernatant following treatment with CA vs. vehicle control (Fig. 4b), with no significant difference observed in phosphate concentration following incubation with solutions of either EDTA or STS vs. vehicle control. Over the course of 6-h, cumulative [phosphate] remained proportional to treatment time. At each time point, CA treatment resulted in greater release of phosphate from the calcified matrix (CA > EDTA > STS), corroborating the pharmaceutical dissolution data. For EDTA and STS, a significant difference in the cumulative phosphate concentration vs. vehicle control was not detected until 3- and 5-h, respectively. After each hour of incubation with fresh chelator solution, the additional amount of phosphate detected in the supernatant remained consistent and indicated that the amount of chelator was the limiting factor in the dissolution process. If the chelators were applied at near-saturated concentrations—as opposed to predicted dermal concentrations—both CA and EDTA completely remove all matrix calcification within 5 min, with maximum phosphate concentrations observed for both chelators (Fig. S3).

Staining of the matrix with ARS confirmed that CA was most effective at removing calcification (Fig. 4c). Of the three chelators tested, CA was the only compound to show a significant decrease in the amount of ARS staining *cf.* vehicle control. After 3-h of repeated hourly treatment with CA, a significant decrease in staining was detected *cf.* vehicle control (75 ± 10 vs. 98 ± 5%, respectively), and at 6-h, only ~ 41% of calcification remained (Fig. 4d). No significant difference in ARS staining was detected between treatments of EDTA and STS *cf.* vehicle control (Fig. 4c).

## Discussion

Here we show that the polycarboxylic acids CA and EDTA are superior in dissolving HAp tablets in an in vitro pharmacological dissolution model of calcinosis cutis, to STS, and further establish that chelators do not induce significant toxicity or inflammation in skin model systems. We further demonstrate, using a 3D skin model, that application of topical chelators fails to disrupt the organisation of the epidermis or cause irritancy. When applied at relative dermal concentrations, CA and EDTA were able to dissolve both HAp tablets and remove a calcified matrix, with CA performing better due to its higher achievable local concentration.

Polycarboxylic acids are well known to chelate metal ions with high affinity. This has led to their use in a range of applications, including as anti-coagulants^27^, in dentistry^20^ and even as cell dissociation reagents^28,29^. When HaCaT cells were exposed to each compound in solution, the concentration-dependent cytotoxicity profiles correlated with their relative ability to dissolve HAp (STS < CA < EDTA). Whilst STS (0.001–10 mM) did not induce observable cytotoxic effects, both CA and EDTA showed some cytotoxic effects at the highest concentration of 10 mM. However, this was related to sequestration of Ca^2+^ ions; the cytotoxic effects of both compounds were abolished when media was supplemented with an equivocal concentration of CaCl_2_ (10 mM). However, even with additional CaCl_2_, cell viability remained significantly lower for cells treated with EDTA; this reduced viability may be due to the acidification of the media as at pH 7.4; EDTA exists as a trivalent ion (HY^3−^) whilst formation of calcium disodium EDTA would displace hydrogen ions (H^+^) to the media.

None of the chelators at concentrations < 10 mM induced an acute irritation response in 2D HaCaT cells. We observed anti-inflammatory effects when cells were treated with CA or EDTA (10 mM); this has been previously reported^30^. Production and secretion of IL-8 is tightly-regulated by intracellular Ca^2+^ ([Ca^2+^]_i_)^31^ and activation of nuclear factor of activated T-cells (NFAT), a transcription factor regulated through the calcium-dependent calmodulin/calcineurin complex^32^. Both calcineurin and NFAT are ubiquitously expressed in keratinocytes and fibroblasts^33^. Therefore, a significant reduction in [Ca^2+^]_i_ could inhibit the activation of the calcineurin/ NFAT pathway, so reducing IL-8 secretion.

When testing each compound in 3D recombinant human epidermal models (RHE; SkinEthic™), no compound was classed as irritant, despite much higher testing concentrations^34^. Hence, RHE models give a more accurate prediction of in vivo pharmacological and toxicological effects^35^. Such models have been evaluated for their ability to correctly predict topical skin irritation, with a predictive ability of ≥ 90%. Importantly, RHE models contain all viable (*stratum basale*, *stratum spinosum*, and *stratum granulosum*) and non-viable (*stratum corneum*) cell layers, with features similar to human epidermis. As the *stratum corneum* is the major barrier limiting compound permeation, it is not surprising to observe differences in toxicity measures across 2D and 3D systems. Using SkinEthic™ models, acute skin irritation was not expected using near-saturated solutions of STS, CA and EDTA, prepared at pH 5.5 (the pH of skin). These findings therefore predict the relative concentration of each compound which can safely be applied to skin (CA > STS > EDTA). Previous in vivo studies corroborate the topical use of STS and CA at concentrations indicated in this study; topical STS has been used at concentrations of 25% (w/v) for the treatment of ectopic calcifications, with only 8% of cases documenting skin irritation^16^. Similarly, CA has been used at concentrations of up to 25% for applications targeting rejuvenation of photoaged skin^36–38^. In these cases, the compounds were applied daily for several months. Less information is available on the topical application of EDTA (7.5% w/v). Whilst used cosmetically at very low concentrations (0.001–0.8%)^18^, at higher concentrations, EDTA and its disodium dehydrate may cause contact dermatitis^39^.

We used RHE models to predict relative percutaneous absorption through skin, as compound absorption over 6-h is comparable to that for human and pig skin^40^. When applied at near-saturated concentrations (finite-dose), compound absorption was linear, with the relative amount of compound detected in the supernatant correlating with the initial concentration applied to the epidermal surface. Histological examination also confirmed that chelators did not disrupt the integrity of the epidermal barrier over this time period. However, impact of topical application may only truly be assessed in vivo. This data was used to predict compound concentrations available to dissolve calcification. The dissolution performance of each chelator was then tested at these predicted dermal concentrations—using two different in vitro models of calcinosis—as a predictive measure of relative therapeutic efficacy. The first model compared dissolution of HAp over time at physiological conditions (37 °C), whereas the second model compared rate of dissolution with repeated application of chelator solutions using a calcified ECM. As calcinosis consists of both inorganic crystalline material (HAp) and organic material (approximately 50%)^1^, these models provide a broad representation of physiological calcinosis. Importantly, both models highlighted CA as the most effective chelator, solubilising more HAp and removing more ECM calcification than either EDTA and STS at predicted dermal concentrations. Interestingly, EDTA proved more effective than STS, despite being prepared at much lower concentrations. Overall, this study highlights the potential use of polycarboxylic acids—particularly CA—in topical therapies targeting subcutaneous calcification. Early phase clinical studies in healthy controls (to assess tolerance) and in patients with SSc-related calcinosis are now required, as a next step in identifying a safe and effective treatment for this painful and debilitating problem.

## Supplementary Information

Supplementary Information.

## Data Availability

The datasets used and/or analysed during the current study available from the corresponding author on reasonable request.

## Abbreviations

CA: Citric acid
EDTA: Ethylenediaminetetraacetic acid
Hap: Hydroxyapatite
SSc: Systemic sclerosis
STS: Sodium thiosulfate
