# Supplementary material for: Citric acid is more effective than sodium thiosulfate in chelating calcium in a dissolution model of calcinosis
Source: Sci Rep. 2024 Dec 28;14:30645. doi: 10.1038/s41598-024-65761-3 (PMC11681028; doi:10.1038/s41598-024-65761-3)

**Citric acid is more effective than sodium thiosulfate in chelating calcium in a dissolution model of calcinosis**

**Running head:** Chelation for *cutis calcinosis*

**Supplementary**

**Materials & Methods**

**Preparation of hydroxyapatite tablets**

Briefly, HAp was prepared following dropwise addition of phosphoric acid [H_3_PO_4_] (85%, 0.554 mL, 8.1 mmol) into a solution of calcium hydroxide [Ca(OH)_2_] (1.00 g, 13.5 mmol) dissolved in 40 mL of water solution. The pH was continuously monitored to ensure that the pH of the solution stayed above pH ~~>~~ 9.5 following dropwise addition of phosphoric acid. The precipitate was washed with de-ionised water using multiple cycles of centrifugation/resuspension, frozen and lyophilised. Tablets were manufactured using a single punch press with manual feeding, yielding the following specifications: mass = 0.22 ± 0.007 g; diameter = 9.55 ± 0.01 mm; height = 2.4 ± 0.2 mm; hardness (P_max_) = 6.6 ± 1.5 kg (Tablet hardness tester TBF 1000, Copley Scientific, U.K); Δm_Friability_= < 1%, 20 randomly selected tablets after 100 cycles (Friability tester, Copley Scientific, U.K). No disintegration was observed after 15 mins in water at 35°C, and none of randomly selected tablets used in the described experiments disintegrated (Tablet Disintegration Tester ZT 31, Copley Scientific, U.K).

**Pharmaceutical dissolution of hydroxyapatite tablet**

Dissolution of HAp tablets were tested with a Dissolution Tester DIS 8000 (Copley Scientific, UK). Each vessel was filled with 900 mL of 10 mM HEPES buffer (pH 7.4), pre-warmed to 37°C, and stirred with paddles (60 rpm). One HAp tablet per vessel was added and incubated for 10 min, followed by addition of 100 mL of chelator concentrate (total volume, 1000 mL). The chelator concentrates (STS, CA or EDTA) were prepared at 10X concentrations in 10 mM HEPES, adjusted to pH to 7.4. A vehicle control (10 mM HEPES) was used to account for any non-specific dissolution. Samples (10ml) were collected after 1-hour and filtered (0.22 μm filter; Whatman; Little Chalfont, UK). Calcium ions were quantified using inductively coupled plasma optical emission spectroscopy (ICP-OES: Thermo iCap 6300). Calcium concentrations were expressed as HAp based on the following relationship: 1 mol calcium = 100.5 g HAp.

For dissolution studies at predicted dermal concentrations (Figure 4a), the following changes were made to the method described above. Each vessel was filled with 400 mL of 1 mM HEPES buffer (pH 7.4). The chelator concentrates were prepared at 10X concentrations [STS (2.6 g/L), CA (4.3 g/L) and EDTA (0.5 g/L)] in 1 mM HEPES (pH 7.4). A vehicle control (1 mM HEPES) was used to account for any non-specific dissolution. Samples (10 mL) were collected every hour over a 5-hour time course. After each sample, fresh chelator solutions (1X) were added to maintain receptor volume.

**Ion chromatography**

Briefly, samples were run on a Dionex^TM^ ICS5000 (ThermoFisher) using an IonPac^TM^ AS11-HC hydroxide-selective anion-exchange capillary column (injection volume: 0.4 µl; flow rate: 0.015 ml/min).  Anions were detected using an electrical conductivity detector and the peak area of a sample was compared to a calibration curve for citrate, thiosulphate and EDTA (0-20 mg/L) to determine its concentration. Chromatogram integration was performed using Chromeleon 7.1.

**Figure S1: Irritation response of HaCaT cells following treatment with sodium thiosulfate (STS), citric acid (CA) and ethylenediaminetetraacetic acid (EDTA).** HaCaT cells were treated with increasing concentrations of calcium chelators for 3- and 24-hrs and the relative expression of pro-inflammatory genes (IL-1α and IL-8) and protein secretion (IL-8) was determined using (a) RT-qPCR and (b) ELISA, respectively. LPS was used as a positive control (10 µg/ml). Relative gene expression is normalised to GAPDH expression. All data shown is relative to a control (vehicle) and presented as mean ± stdev (N=3); *, P < 0.05 *cf*. control.

**Figure S2: Predicted skin irritation response of different calcium chelators using the SkinEthic^TM^ Skin Irritation Test (SIT).** SkinEthic^TM^ models (day 17) were treated with increasing concentrations of (a) STS [200 – 1200 mM], (b) CA [200 – 1600 mM], or (c) EDTA [50 – 200 mM] at pH 5.5 (10 mM HEPES buffer) for 42 minutes at room temperature. Three controls were included: PBS (negative), 5% SDS (positive) and 10mM HEPES buffer (vehicle). Models were then incubated for 42 hours following treatment and cell viability was assessed using the MTT assay. Data is relative to the negative control and presented as mean ± stdev (N=3). The dotted line represents 50% reduction in viability – the threshold for categorising a compound as an irritant (Category 2). (a) STS, (b) CA, (c) EDTA.

**Figure S3: Dissolution of matrix calcification following incubation with calcium chelators at near-saturated concentrations.**

(A) Representative images of Alizarin Red S staining of calcified matrix following 5-minute incubation with different chelator solutions at near-saturated concentration: STS (1200 mM), CA (1600 mM) and EDTA (200 mM). Three controls were included: positive (untreated calcified matrix), negative (matrix deposited by cells cultured in normal media), and vehicle control (HEPES 10 mM). (B) The amount of Alizarin Red stain following 5 minutes chelator treatment of the calcified matrix is quantified relative to untreated calcified matrix (osteogenic). (C) The concentration of phosphate in the supernatant after 5 minutes chelator treatment of the calcified matrix. Data is presented as mean ± stdev (n = 3); *, P < 0.05 *cf.* vehicle; ^#^, P < 0.05 *cf.* STS.

**Supplementary Figure S1**


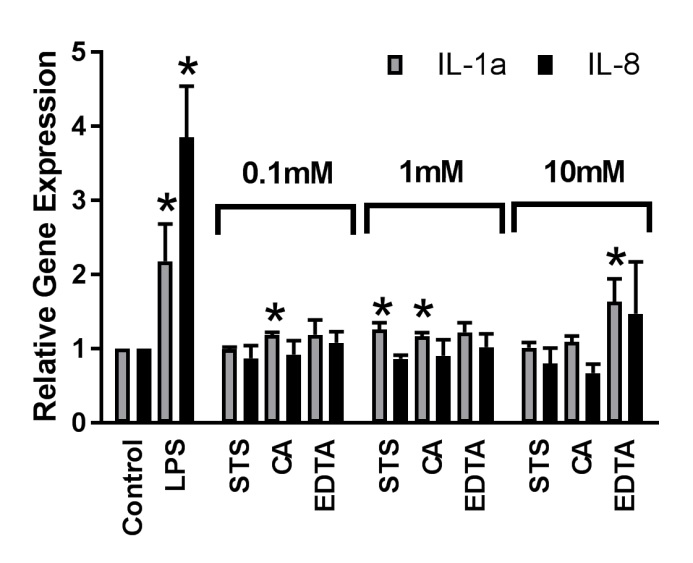


**a**


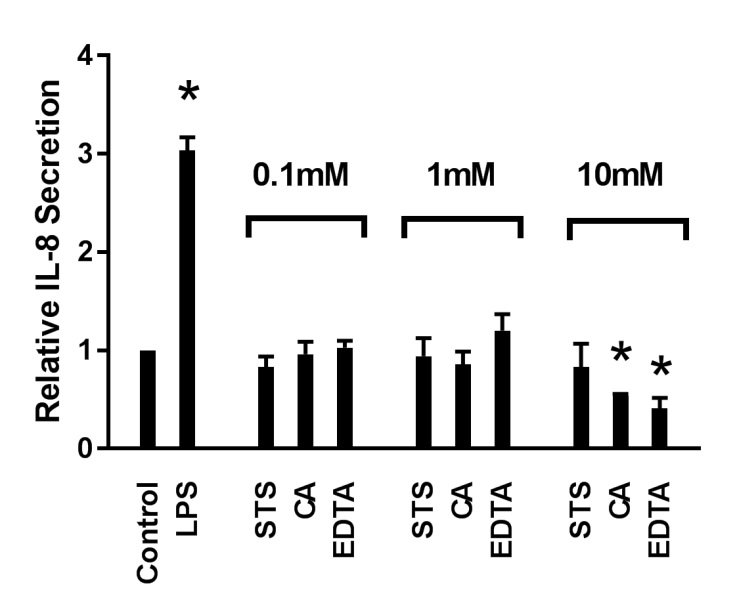


**b**

**Supplementary Figure S2**


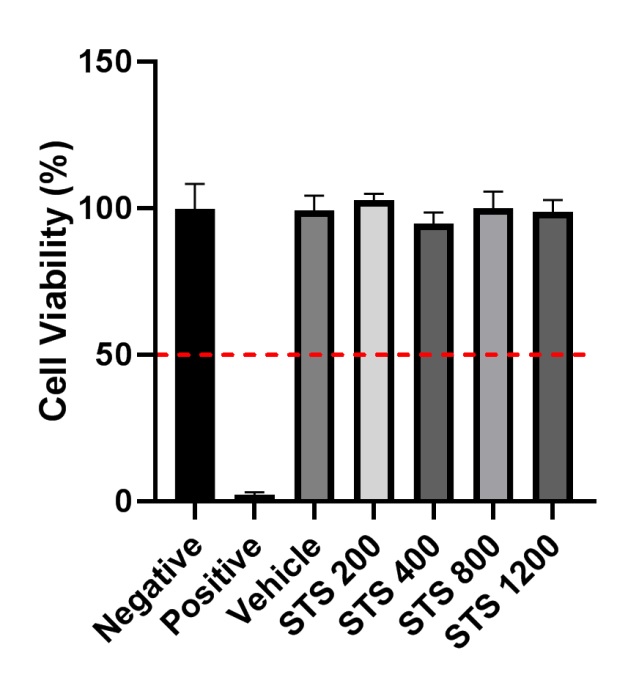

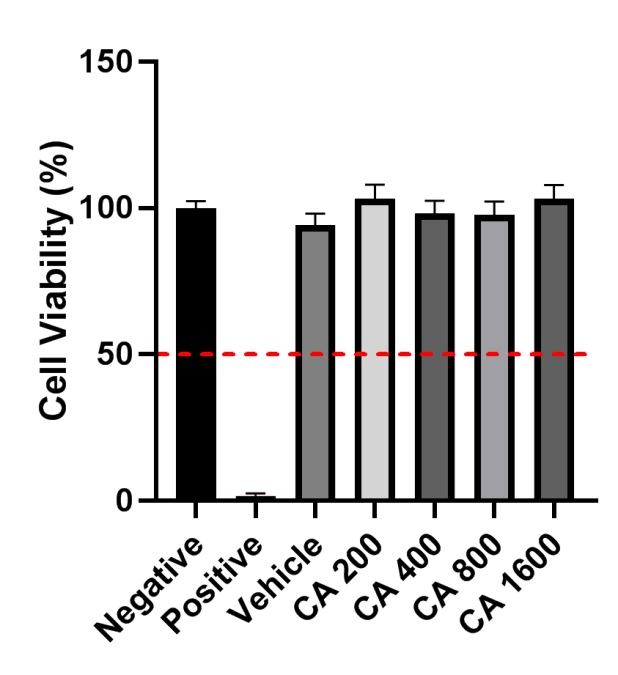

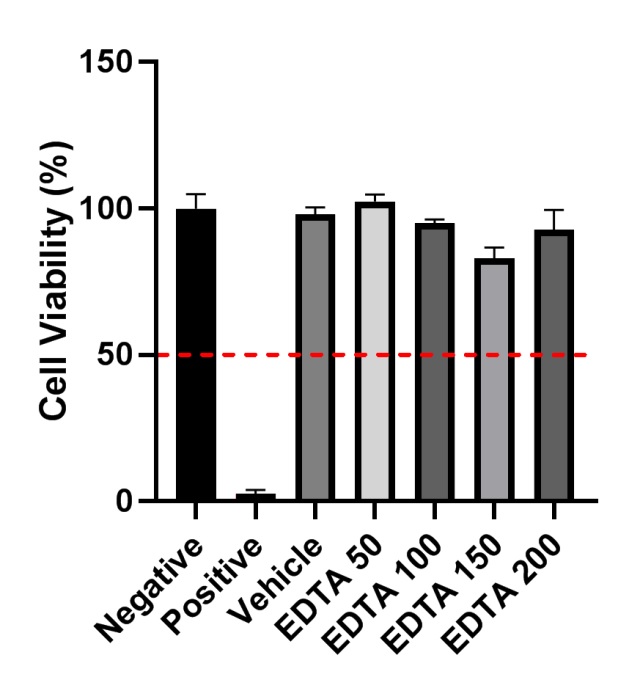


**a**

**b**

**c**

**Figure S3**


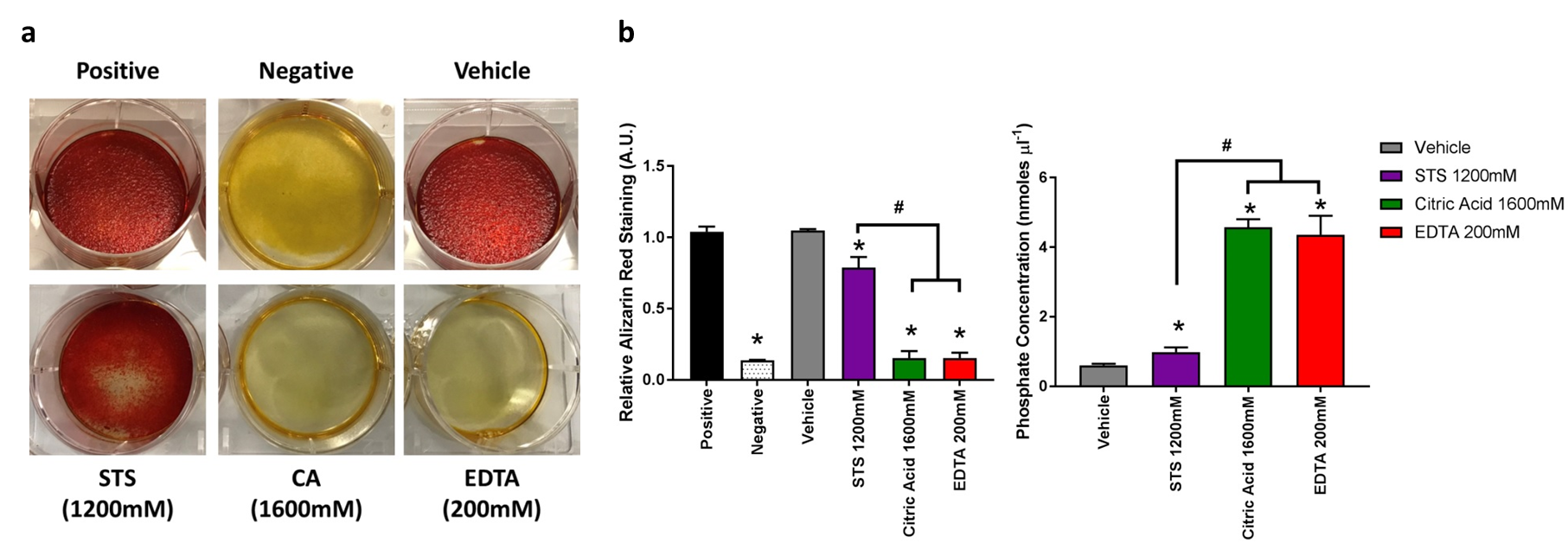

Supplement: Supplementary file 1 — Supplementary Information. [file 41598_2024_65761_MOESM1_ESM.doc]
